# Supplementary material for: Genetic and Epigenetic Regulation of the Smoothened Gene (SMO) in Cancer Cells
Source: Cancers (Basel). 2020 Aug 8;12(8):2219. doi: 10.3390/cancers12082219 (PMC7464114; doi:10.3390/cancers12082219)
Supplement: Supplementary file 1 [file cancers-12-02219-s001.pdf]

cancers

Supplementary Materials

Genetic and Epigenetic Regulation of the Smoothened Gene (SMO) in Cancer Cells

Hong Lou, Hongchuan Li, Andrew R. Huehn, Nadya I. Tarasova, Bahara Saleh, Stephen K. Anderson and Michael Dean

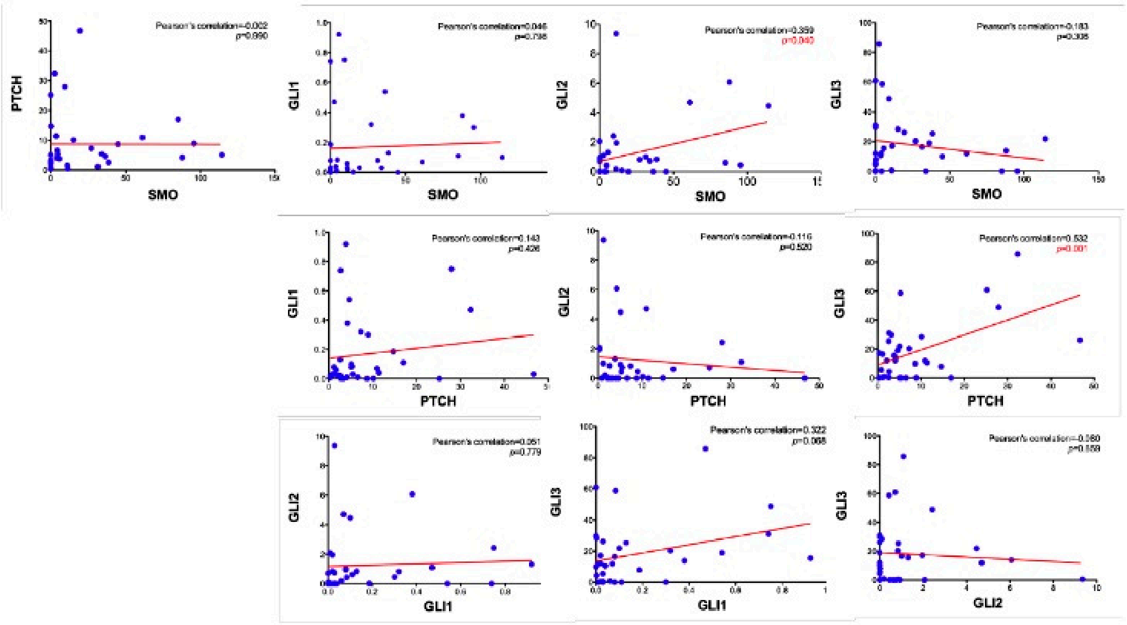

Figure S1. Pearson's correlation analysis of gene expression levels of SMO, PTCH, GLI1, GLI2, and GLI3 in cancer cells. Pearson's correlation coefficients were performed to estimate the correlations of mRNA expression levels using the JMP program.

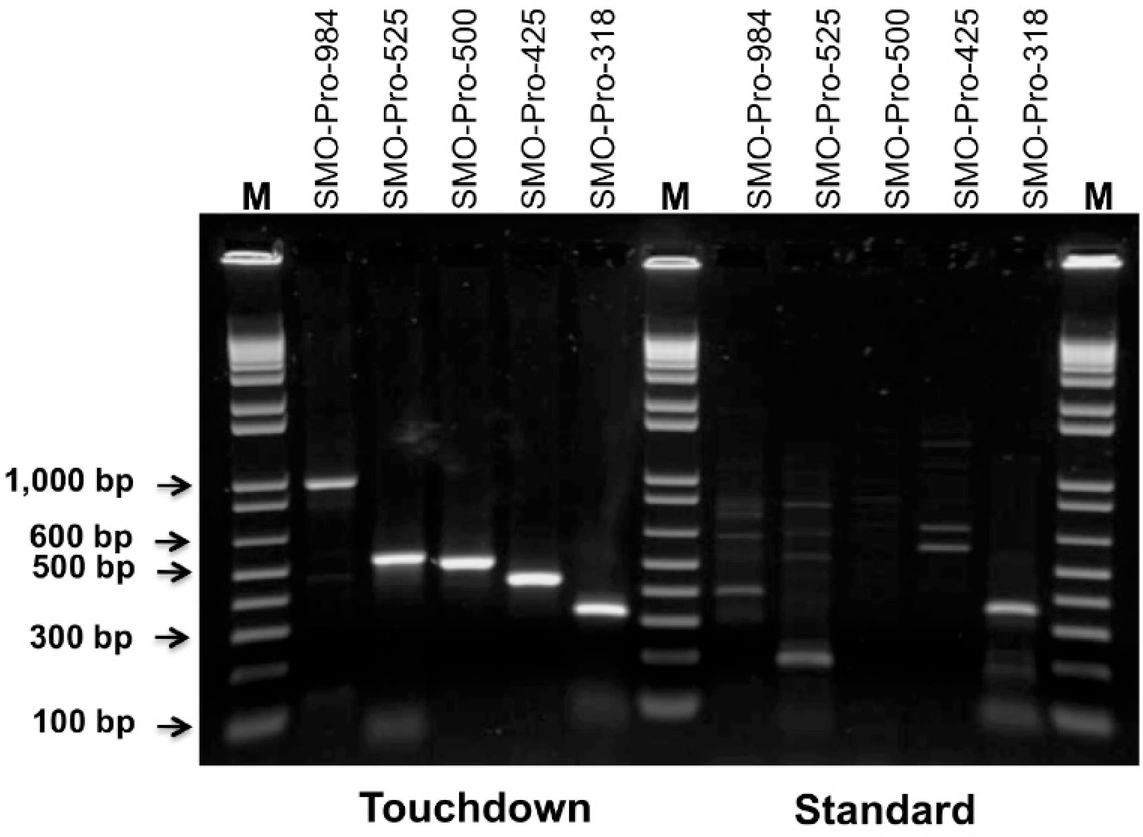

Figure S2. Comparison of PCR conditions to amplify SMO promoter sequence in BT549 DNA. The figure showed PCR products from the five different primer sets used in the 5′-deletion assay to amplify 984bp, 525bp, 500bp, 425bp, and 318bp fragments of the SMO promoter (M&M section), respectively. Equal amounts (15 µl) were loaded on a 1.0% agarose gel. Left panel showed PCR results from the touchdown PCR, and right panel showed PCR results from the standard PCR program. Lane M, molecular weight marker (1kb plus DNA ladder).

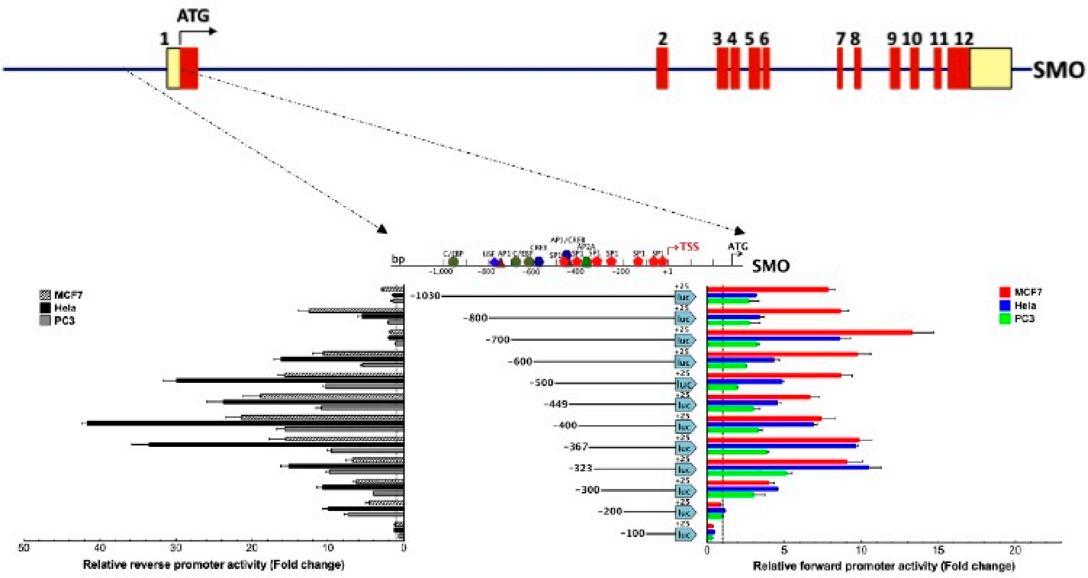

Figure S3. Identification of SMO promoters by 5′ truncation analysis. A schematic representation of the SMO bidirectional element is shown with the potential transcription factor binding sites. Serial

deletions at the 5′ end of the promoter fragment of SMO are shown. The luciferase activity of pGL3 constructs containing fragments cloned in either the reverse (left panel) or forward (right panel) orientation is shown as fold-increase of corrected light units relative to an empty pGL3 vector control [48]. Values represent the mean±SEM of at least three independent experiments.

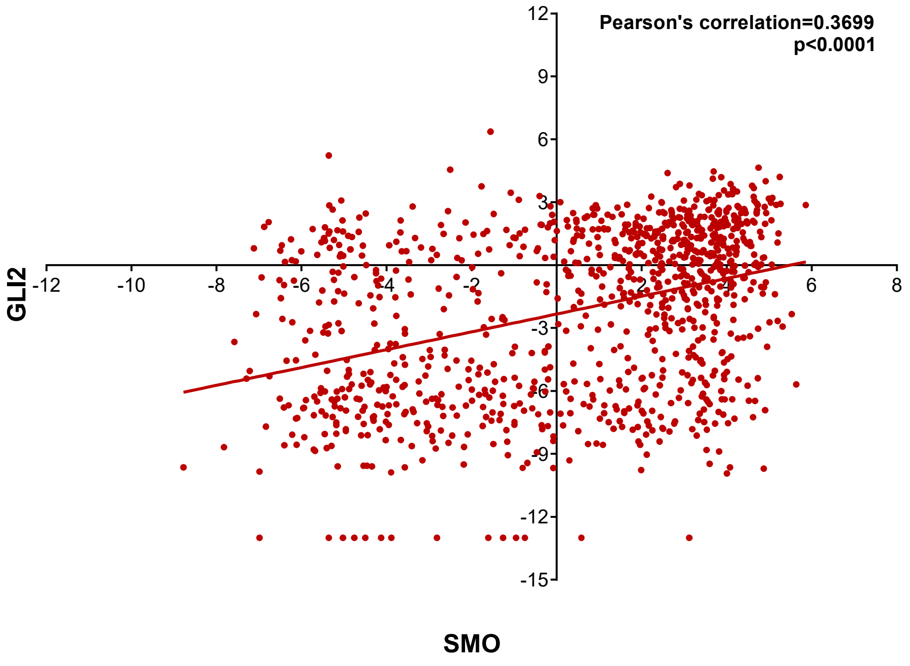

Figure S4. Pearson's correlation analysis of gene expression levels of *SMO* and *GLI2* in 1119 cancer cells from the Cancer Cell Line Encyclopedia (https://portals.broadinstitute.org/ccle). Pearson's correlation coefficients were performed to estimate the correlation of mRNA expression level from the cell linesusing the JMP program.

Table S1. Sequence of primers used in this study.

Amplicon	Direction	Primer Name	Sequence (5'->3')	Length
5'-deletion reporter assay				
	Forward	SMO-Pro-959-Fwd	AATGAGCAGAGTGGGGAAGA	984 bp
	Forward	SMO-Pro-500-Fwd	ACCTCAGACCAAGCAAGGTGC	525 bp
	Forward	SMO-Pro-475-Fwd	CGAGTCTCTCCTTGCAGGTC	500 bp
	Forward	SMO-Pro-400-Fwd	GGTCACCAGATCCCCCTAG	425 bp
	Forward	SMO-Pro-293-Fwd	CCCTCCCCCAGCCTCGGCG	318 bp
	Reverse	SMO-Pro+25-Rev	TCGCAATCCAAGTTGTCTTCAGCCC	
3'-deletion assay				
	Forward	SMO-Pro-500-Fwd	ACCTCAGACCAAGCAAGGTGC	
	Reverse	SMO-Pro+52-Rev	GGATGCACGACTCCCCAAGC	552 bp
	Reverse	SMO-Pro+25-Rev	TCGCAATCCAAGTTGTCTTCAGCCC	525 bp
	Reverse	SMO-Pro+15-Rev	AGTTGTCTTCAGCCCTAGGAGACC	515 bp
MSP				
SMO ummethylated	Forward	SMO-MSP-UF	TTTTTGTAGTTTAATATGGGTTTTGG	132 bp
	Reverse	SMO-MSP-UR	AAAAACAACTCAAACAACCTATACACC	
SMO methylated	Forward	SMO-MSP-MF	TTTTCGTAGTTTAATATGGGTTTCGG	132 bp
	Reverse	SMO-MSP-MR	GAAAACGACTCAAACGACCTATACG	
Actin-beta	Forward	ACTB Forward	TGGTGATGGAGGAGGTTTAGTAAGT	133 bp
	Reverse	ACTB Reverse	AACCAATAAAACCTACTCCTCCCTTAA	
BSP				
SMO CpG Island 1	Forward	Smobis1*	GAYGATTTTAGATTAAGTAAGGTGTT	464 bp
	Reverse	Smobis3*	TTYGTGTATTTTAGAGAGTTTAG	
SMO CpG Island 2	Forward	SMOBSP-CpG2-F1	GGGTTTTTTAGGGTTGAAGATAATT	359 bp
	Reverse	SMOBSP-CpG2-R	CAACAACAACAACAACAACAAC	
SMO CpG Island 3	Forward	SMOBSP-CpG3-F1	GGGTTGTTGTTGTTGTTGTTGT	351 bp
	Reverse	SMOBSP-CpG3-R	TCCAAAAATACCCCCATCTTAC	

Table S2. Expression level of hedgehog pathway genes in NCI-60 tumor cell lines.

Cell Lines	SMO	PTCH1	GLI1	Tissue Type
BT549	15.80	3.51	2.31	Breast
HS578T	28.31	3.34	4.28	Breast
MB-231	0.00	9.84	0.01	Breast
MCF7	0.00	8.70	0.30	Breast
MDA-MB-468	33.40	12.54	0.31	Breast
T47D	10.52	1.29	0.02	Breast
SF-295	10.51	3.69	1.20	Central Nervous System
SF-539	28.42	1.47	1.03	Central Nervous System
SF-268	54.06	5.61	0.26	Central Nervous System
SNB-75	16.22	8.57	1.49	Central Nervous System
SNB-19	54.04	5.98	0.08	Central Nervous System
U251	26.99	3.65	0.28	Central Nervous System
COLO 205	0.00	19.50	0.08	Colon
HCC-2998	5.80	12.19	0.35	Colon
HCT-116	11.05	3.73	0.81	Colon
HCT-15	0.00	25.31	0.57	Colon
HT29	0.00	7.37	0.14	Colon
KM12	0.00	7.60	0.30	Colon
SW-620	10.89	6.59	0.12	Colon
CCRF-CEM	7.74	6.98	0.30	Leukemia
HL-60	0.68	16.05	0.11	Leukemia
K-562	0.05	9.08	0.42	Leukemia
MOLT-4	50.59	0.77	0.65	Leukemia
RPMI-8226	0.00	5.87	0.52	Leukemia
SR	0.00	2.59	0.08	Leukemia
LOXIMVI	0.00	12.43	0.32	Melanoma
M14	27.70	28.70	0.11	Melanoma
MALME-3M	82.34	10.22	0.02	Melanoma
SK-MEL-2	1.46	12.54	0.42	Melanoma
SK-MEL-28	36.82	7.68	0.09	Melanoma
SK-MEL-5	39.20	2.32	0.09	Melanoma
UACC-257	23.80	11.61	0.10	Melanoma
UACC-62	4.55	6.84	0.09	Melanoma
MB-435	1.62	6.94	0.36	Melanoma
A549	27.97	10.24	4.38	Lung
EKVX	55.94	15.70	1.93	Lung
HOP62	58.73	7.86	0.04	Lung
HOP92	0.24	11.37	0.03	Lung
NCI-H23	45.91	7.93	7.07	Lung
NCI-H322M	22.33	8.18	0.07	Lung
NCI-H460	14.67	7.97	0.24	Lung
NCI-H522	95.11	24.67	19.59	Lung
NCI-H226	39.24	2.18	0.34	Lung
IGROV1	26.99	15.06	0.21	Ovarian
OVCAR-4	11.55	11.49	0.30	Ovarian
OVCAR-3	101.82	11.08	0.27	Ovarian
OVCAR-5	43.24	3.00	1.42	Ovarian
OVCAR-8	20.87	1.84	0.22	Ovarian
SK-OV3	118.43	3.89	6.67	Ovarian
NCI/ADR-RES	45.60	2.81	0.74	Ovarian
DU145	8.62	4.39	0.22	Prostate
PC3	7.06	1.53	0.02	Prostate
786-0	14.89	15.03	0.72	Kidney
A498	25.93	5.15	0.37	Kidney
ACHN	25.12	52.13	0.26	Kidney
CAKI-1	49.40	7.40	0.12	Kidney
RXF-393	28.95	13.10	0.30	Kidney
SN12C	96.86	5.04	0.09	Kidney
TK-10	51.34	11.78	0.72	Kidney
UO-31	9.16	1.05	0.04	Kidney

Abbreviation: ND, not detectable; *: Relative mRNA Expression = (Target gene/18sRNA) ×10e6.

© 2020 by the authors. Licensee MDPI, Basel, Switzerland. This article is an open access article distributed under the terms and conditions of the Creative Commons Attribution (CC BY) license (http://creativecommons.org/licenses/by/4.0/).
